# Machine learning-based early prediction of severe pneumonia risk in hospitalized leukemia patients: a dual-center retrospective cohort study

**DOI:** 10.3389/fcell.2026.1943797

**Published:** 2026-09-18

**Authors:** Jian Huang, Qinliang Wu, Yixin Zhang, Kai Wang, Yuanyuan Wang, Chaogui Tang, Chang Li, Ning Lin

**Affiliations:** 1 Department of Medical Laboratory, The Affiliated Huai’an No. 1 People’s Hospital of Nanjing Medical University, Huai’an, Jiangsu, China; 2 Department of Medical Laboratory, Central Theater Command General Hospital of Chinese People’s Liberation Army, Wuhan, China; 3 Department of Biostatistics, Center for Global Health, School of Public Health, Nanjing Medical University, Nanjing, Jiangsu, China; 4 Department of Rheumatology, The Affiliated Huai’an No. 1 People’s Hospital of Nanjing Medical University, Huai’an, Jiangsu, China

**Keywords:** external validation, leukemia, machine learning, nomogram, prediction model, severe pneumonia, SMOTE

## Abstract

**Background:**

Severe pneumonia is a major infectious complication in patients with leukemia and may rapidly progress to respiratory failure or septic shock. Disease- and treatment-related immunosuppression increases susceptibility, yet population-specific early risk-stratification tools are limited.

**Objectives:**

To develop and externally validate a machine learning model for predicting severe pneumonia in hospitalized patients with leukemia.

**Methods:**

Data from 2,978 leukemia patients at two Chinese hospitals were retrospectively analyzed. The derivation cohort was divided into training and internal test sets; SMOTE was applied only to the training set. LASSO and SVM–RFE were used for predictive feature selection, eight machine learning algorithms were compared, and RCS was used for exploratory association analysis. The final model was presented as a nomogram.

**Results:**

Seven predictors were retained: age, history of myelosuppression, acute lymphoblastic leukemia (ALL) subtype, cardiac insufficiency, atrial fibrillation, red blood cell distribution width–standard deviation (RDW-SD), and prothrombin time activity. Logistic regression showed the best generalizability, with an area under the curve (AUC) of 0.801 in external validation. Sensitivity analyses showed similar discrimination with multiple imputation and complete-case analysis.

**Conclusion:**

We developed and externally validated an interpretable model for severe pneumonia in hospitalized patients with leukemia. Because direct programmed-cell-death biomarkers were not measured and SMOTE substantially altered training prevalence, mechanistic interpretation and absolute-risk calibration require caution. Prospective recalibration and clinical-impact evaluation are needed before routine use.

## Introduction

Sepsis is defined as life-threatening organ dysfunction caused by a dysregulated host response to infection (Singer et al., 2016). Severe pneumonia is an important infectious complication that can progress to respiratory failure, septic shock, and multiorgan dysfunction. Experimental and clinical literature has implicated regulated cell-death pathways, including pyroptosis and ferroptosis, in severe infection and sepsis-associated organ injury (Wen et al., 2022; Liu et al., 2022). However, these pathways were not directly measured in the present cohort and are considered biological context rather than study endpoints.

Patients with leukemia are particularly vulnerable to severe respiratory infection because both the underlying malignancy and antileukemic treatment can impair innate and adaptive immunity (Arruga et al., 2020; Ehrlich et al., 2026). During intensive chemotherapy, neutropenia, mucosal barrier injury, corticosteroid exposure, and other treatment-related immune deficits can increase susceptibility to lower respiratory tract infection and infectious complications (Ehrlich et al., 2026; Ullah et al., 2025).

The etiology of pneumonia in immunocompromised patients is heterogeneous and may include conventional bacteria, invasive fungi, Pneumocystis jirovecii, and respiratory viruses (Ullah et al., 2025). Preclinical literature links severe infection to inflammatory cell-death pathways such as pyroptosis and ferroptosis (Wen et al., 2022; Liu et al., 2022), but routine electronic medical records do not directly establish activation of these pathways. Accordingly, any mechanistic interpretation of routinely measured clinical biomarkers in this study is hypothesis-generating.

Early risk stratification of severe pneumonia in hospitalized patients with leukemia remains challenging. The Pneumonia Severity Index (PSI) and CURB-65 were developed and validated primarily in general community-acquired pneumonia populations (Fine et al., 1997; Lim et al., 2003), and their transportability to patients with hematologic malignancies and treatment-related immunosuppression is uncertain. Leukemia-specific factors such as myelosuppression, disease subtype, cardiovascular comorbidity, and coagulation abnormalities may provide additional predictive information; coagulation abnormalities are also well described in acute leukemia (Hisada et al., 2024).

Machine learning (ML) can integrate multidimensional clinical data and has been widely evaluated for infection and sepsis prediction (Fleuren et al., 2020). For clinical prediction, however, discrimination alone is insufficient: transparent feature selection, calibration assessment, external validation, and interpretable reporting are important for clinical translation. The TRIPOD + AI statement provides updated reporting guidance for prediction models developed with regression or machine-learning methods (Collins et al., 2024).

Therefore, we conducted a dual-center retrospective study to develop and externally validate a machine learning-based prediction model for severe pneumonia in hospitalized patients with leukemia. Our objective was to identify a parsimonious set of routinely available admission variables that could support early risk stratification while clearly separating predictive associations from unmeasured biological mechanisms.

## Methods

### Study design and population

This multicenter retrospective cohort study included patients from two Chinese institutions: The Affiliated Huai’an No. 1 People’s Hospital of Nanjing Medical University and Central Theater Command General Hospital of Chinese People’s Liberation Army. The derivation-center records used in this analysis covered 1 January 2015, to 31 May 2025, consistent with the cohort reported in the Results. Clinical data were extracted retrospectively from electronic medical records (EMRs). The primary outcome was severe pneumonia occurring during hospitalization, defined using the 2019 ATS/IDSA major/minor criteria; it was not treated as equivalent to Sepsis-3-defined sepsis (Singer et al., 2016; Metlay et al., 2019).

### Inclusion and exclusion criteria

Patients were eligible for inclusion if they met the following criteria:

1. Confirmed diagnosis of leukemia [acute myeloid leukemia (AML), acute lymphoblastic leukemia (ALL), chronic myeloid leukemia (CML), or chronic lymphocytic leukemia (CLL)] identified through comprehensive diagnostic evaluation including bone marrow aspiration and biopsy, flow cytometric immunophenotyping, and cytogenetic/molecular genetic studies;
2. Hospitalized in the hematology department with a minimum length of stay of 48 h;
3. Availability of complete medical documentation encompassing baseline demographic and clinical characteristics, treatment information, clearly defined outcome data, and the laboratory measurements required for analysis. Predictors used for modeling were restricted to measurements obtained within 24 h of hospital admission and before the occurrence of severe pneumonia.

The exclusion criteria were:

1. pneumonia or severe pneumonia present at admission;
2. pneumonia occurring within 48 h of admission (possibly community-acquired pneumonia, CAP);
3. concurrent other malignancies;
4. HIV infection or other severe immunodeficiency diseases;
5. missing data exceeding 30% for key predictor variables.

### Cohort selection and validation

Initially 2,561 patients from the Affiliated Huai’an No. 1 People’s Hospital of Nanjing Medical University and 585 patients from the Central Theater Command General Hospital of Chinese People’s Liberation Army were identified. Following the application of selection criteria, the final study cohort comprised 2,429 patients from The Affiliated Huai’an No. 1 People’s Hospital of Nanjing Medical University, who were utilized for model development, training, and internal validation. An additional 549 patients from Central Theater Command General Hospital of Chinese People’s Liberation Army constituted an independent external validation cohort to assess model generalizability and performance in a distinct patient population.

### Ethical considerations

This study was conducted in accordance with the Declaration of Helsinki. The research protocol for retrospective analysis of previously collected clinical records received approval from the institutional review boards of both participating institutions (Ethics approval numbers: KY-2025-144-01 and 2026-003-01). Because the study analyzed de-identified historical records and involved no prospective intervention, both ethics committees waived the requirement for written informed consent. The approval dates therefore postdated part of the clinical data-collection period, reflecting the retrospective approval of analysis of existing records.

### Clinical features and data processing

Clinical variables were extracted from EMRs, including demographic characteristics, clinical features, comorbidities, treatment history, baseline clinical status, and laboratory parameters. All predictors were restricted to variables obtained within 24 h of hospital admission and before the occurrence of severe pneumonia. Candidate variables covered routinely available domains relevant to infection risk and physiologic reserve, including coagulation parameters (prothrombin time activity, INR, D-dimer), liver-related tests (AST, ALT, γ-GGT, ALP, ADA), hematologic indices (RDW-SD, RDW-CV), history of myelosuppression, leukemia subtype, and comorbidities. These routine variables were not considered direct biomarkers of programmed cell-death pathways.

A total of 86 candidate variables were collected (Supplementary Tables S1–S2). These included age, sex, leukemia subtype, treatment history (hematopoietic stem cell transplantation, corticosteroid use, granulocyte colony-stimulating factor administration, blood transfusion), comorbidities (e.g., diabetes, cardiovascular diseases, respiratory diseases, renal insufficiency), baseline clinical status, and comprehensive laboratory parameters.

### Outcome definition

#### Diagnostic criteria for leukemia

Leukemia diagnosis and classification were established according to the World Health Organization (WHO) Classification of Haematolymphoid Tumors, fifth edition (2022). AML was diagnosed by ≥ 20% myeloid blasts in bone marrow or peripheral blood, or by the presence of specific genetic abnormalities (Khoury et al., 2022). ALL required ≥20% lymphoid blasts with characteristic immunophenotype (B-cell or T-cell lineage) (Alaggio et al., 2022). CLL was diagnosed by sustained monoclonal B lymphocytosis ≥5 × 10^9^/L for ≥3 months with typical CD5+/CD19+/CD23+ immunophenotype (Hallek et al., 2018). CML diagnosis required detection of BCR/ABL fusion gene by reverse transcription polymerase chain reaction (RT-PCR) or fluorescence *in situ* hybridization (FISH) (Hochhaus et al., 2020). All diagnoses were confirmed by comprehensive evaluation including morphology, immunophenotyping, cytogenetics, and molecular testing.

#### Definition of severe pneumonia

Severe pneumonia was defined according to the 2019 American Thoracic Society (ATS)/Infectious Diseases Society of America (IDSA) guideline (Metlay et al., 2019). Diagnosis required at least one major criterion (invasive mechanical ventilation or septic shock requiring vasopressors) or at least three minor criteria (respiratory rate ≥30/min, PaO2/FiO2 ≤ 250 mmHg, multilobar infiltrates, confusion, blood urea nitrogen ≥7.14 mmol/L, leukopenia, thrombocytopenia, hypothermia, or hypotension requiring aggressive fluid resuscitation). All cases were radiographically confirmed by new or progressive pulmonary infiltrates. The primary outcome in this study was therefore ATS/IDSA-defined severe pneumonia. Although the septic-shock major criterion identifies a subset with overt septic shock, patients meeting only the minor-criterion pathway should not be assumed to satisfy Sepsis-3 without documented infection-related organ dysfunction; severe pneumonia and Sepsis-3 sepsis are not interchangeable clinical entities (Singer et al., 2016).

### Data preprocessing

Outliers in continuous variables were identified using boxplot-based detection as observations exceeding the quartile boundaries by 1.5 interquartile ranges and were truncated to the corresponding boundary values. Records with missingness exceeding the prespecified 30% threshold for key predictors were excluded according to the eligibility criteria. Remaining missing values were handled using multivariate imputation by chained equations (MICE). Laboratory measurements were retained as continuous variables, and categorical variables were one-hot encoded as required. Preprocessing was separated between the derivation workflow and untouched validation datasets to reduce information leakage. A complete-case sensitivity analysis was performed to evaluate the influence of imputation.

### Class balancing and model development

The 2,429 eligible patients in the derivation cohort were first randomly divided into a training set (70%, n = 1,700) and an internal testing set (30%, n = 729). Severe pneumonia occurred in 4.18% of the training set, indicating substantial class imbalance. SMOTE was then applied exclusively to the training set, increasing the severe-pneumonia proportion from 4.18% to 48.95% to increase minority-class representation during feature selection and model fitting. The analytical sequence was therefore: derivation split → training-set preprocessing/SMOTE → LASSO and SVM-RFE feature selection → model fitting and cross-validation. The internal testing set and the external validation cohort were not resampled and retained their original event prevalence. Because near-equal oversampling markedly changes training prevalence, discrimination and calibration on the unresampled validation cohorts were emphasized, and absolute predicted probabilities should be interpreted cautiously.

### Feature selection

Predictive feature selection was performed within the training workflow using a sequential LASSO–SVM-RFE strategy. LASSO regression was applied first, with the penalty parameter (λ) determined by 10-fold cross-validation; SVM-RFE was then applied to the LASSO-selected features using iterative cross-validation. This predictive feature-selection pathway was distinct from the conventional univariable/multivariable logistic regression and RCS analyses, which were performed as exploratory association analyses. Consequently, variables associated with the outcome in exploratory regression or RCS analyses were not required to enter the final prediction model. Multicollinearity among final candidate predictors was assessed using the variance inflation factor (VIF), with VIF <5 considered acceptable.

### Model development and validation

Eight algorithmic approaches were applied: logistic regression (LR), decision tree (DT), random forest (RF), extreme gradient boosting (XGBoost), support vector machine (SVM), K-Nearest Neighbors (KNN), Light Gradient Boosting Machine (LightGBM), and Naive Bayes (NB). Hyperparameters were optimized using grid search with repeated 10 × 10-fold cross-validation. The reliability and performance of the models were comprehensively evaluated using receiver operating characteristic curve analysis (AUC), sensitivity, specificity, PPV, NPV, F1 score, Brier score, Hosmer-Lemeshow test, and decision curve analysis (DCA). The optimal model was selected based on comprehensive evaluation in both training and external validation sets.

### Model interpretability: nomogram and SHAP analysis

A nomogram was constructed from the final selected logistic regression model to support individualized estimation of severe-pneumonia risk. Model coefficients were transformed into a points-based scoring system. SHAP (SHapley Additive exPlanations) values were used to quantify each predictor’s contribution to model output at the global and patient levels. SHAP values were interpreted as model-attribution measures rather than evidence that a predictor directly activates a specific biological or cell-death pathway.

### Web-based calculator

A Shiny implementation was developed to facilitate calculation from the seven final predictors: age, history of myelosuppression, ALL subtype, cardiac insufficiency, atrial fibrillation, RDW-SD, and prothrombin time activity. The complete logistic equation and coefficients are reported in the Results so that the model can be calculated independently of a hosted platform. No single universal diagnostic cutoff was prespecified; the decision-curve analysis was used to describe threshold-dependent net benefit, and any operational threshold should be selected according to the clinical context. Because hosted applications may be updated or become unavailable, the online implementation should be verified against the published seven-variable equation before clinical use.

### Statistical analysis

Algorithmic development employed the R computational platform (version 4.3.1) with the “caret” package (version 6.0-94). Model discrimination was assessed by receiver operating characteristic curve analysis with bootstrap-derived 95% confidence intervals (1,000 replications). Calibration evaluation incorporated Brier score calculation and the Hosmer-Lemeshow test, and decision curve analysis quantified net benefit across probability thresholds. Normally distributed continuous data were summarized as mean ± standard deviation and compared using Student’s t-test; non-parametric variables were expressed as median (interquartile range) and assessed using the Mann-Whitney U or Kruskal-Wallis test. Categorical data were analyzed using Pearson’s chi-square test. Restricted cubic spline (RCS) analysis was performed as an exploratory assessment of nonlinear associations and was not used to select variables for the final prediction model. Statistical significance was defined as two-sided P < 0.05.

## Results

### Study population and cohort characteristics

The study population comprised hospitalized leukemia patients at The Affiliated Huai’an No.1 People’s Hospital of Nanjing Medical University from 1 January 2015 to 31 May 2025. From an initial cohort of 2,561 patients, 2,429 met eligibility criteria and comprised the derivation cohort. The derivation cohort was randomly divided into a training set (n = 1,700) and internal validation set (n = 729) at a 7:3 ratio. For external validation, an independent cohort of 549 patients was recruited from Central Theater Command General Hospital of Chinese People’s Liberation Army (Figure 1).

**FIGURE 1:**
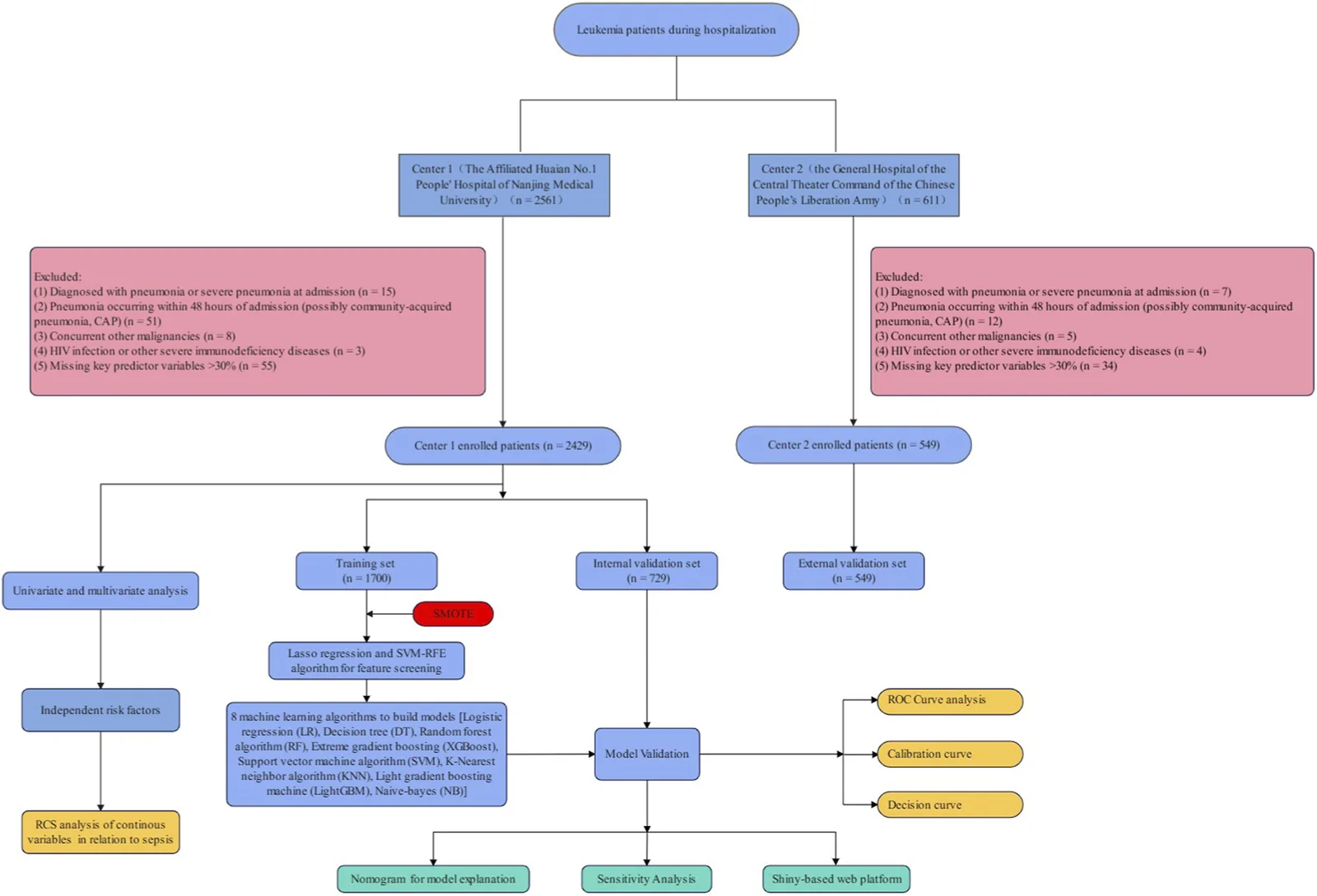
Research flowchart.

### Incidence of severe pneumonia-associated sepsis and baseline characteristics

The overall incidence of severe pneumonia—the primary precipitant of sepsis—among hospitalized patients with leukemia was 3.99% (97/2,429) in the derivation cohort. Comparable rates were observed in the training (4.18%, 71/1,700) and internal validation subsets (3.57%, 26/729). In the external validation cohort (n = 549), the incidence was 4.74% (26/549), indicating consistent event rates across institutions (Table 1).

**TABLE 1:** Comparison of demographic characteristics and clinical characteristics between two centers.

Characteristic	Total (N = 2978)	Center 1 (N = 2429)	Center 2 (N = 549)	p-value
Age (year), median (IQR)	54.00 [36.00; 65.00]	55.00 [36.00; 66.00]	54.00 [35.00; 65.00]	0.056
Gender, n (%)	​	​	​	0.357
Female	1274 (42.78%)	1029 (42.36%)	245 (44.63%)	​
Male	1704 (57.22%)	1400 (57.64%)	304 (55.37%)	​
Malignancy type, n (%)				
Acute myeloid leukemia	​	​	​	0.083
No	1372 (46.07%)	1121 (46.15%)	231 (42.08%)	​
Yes	1606 (53.93%)	1308 (53.85%)	318 (57.92%)	​
Acute lymphoblastic leukemia	​	​	​	0.094
No	2444 (82.07%)	1999 (82.30%)	435 (79.23%)	​
Yes	534 (17.93%)	430 (17.70%)	114 (20.77%)	​
Chronic lymphocytic leukemia	​	​	​	**0.026**
No	2719 (91.30%)	2204 (90.74%)	515 (93.81%)	​
Yes	259 (8.70%)	225 (9.26%)	34 (6.19%)	​
Chronic myeloid leukemia	​	​	​	0.092
No	2398 (80.52%)	1962 (80.77%)	426 (77.60%)	​
Yes	580 (19.48%)	467 (19.23%)	123 (22.40%)	​
Treatment history				
History of hematopoietic stem cell transplantation	​	​	​	0.068
No	2771 (93.05%)	2270 (93.45%)	501 (91.27%)	​
Yes	207 (6.95%)	159 (6.55%)	48 (8.74%)	​
History of corticosteroid use	​	​	​	0.063
No	2807 (94.26%)	2277 (93.74%)	530 (96.54%)	​
Yes	171 (5.74%)	152 (6.26%)	19 (3.46%)	​
History of G-CSF use	​	​	​	0.257
No	2805 (94.19%)	2294 (94.44%)	511 (93.08%)	​
Yes	173 (5.81%)	135 (5.56%)	38 (6.92%)	​
History of blood transfusion	​	​	​	**0.041**
No	2640 (88.65%)	2167 (89.21%)	473 (86.16%)	​
Yes	338 (11.35%)	262 (10.79%)	76 (13.84%)	​
History of myelosuppression	​	​	​	0.075
No	2696 (90.53%)	2210 (90.98%)	486 (88.52%)	​
Yes	282 (9.47%)	219 (9.02%)	63 (11.48%)	​
Comorbidities, n (%)				
Diabetes mellitus	​	​	​	0.067
No	2668 (89.59%)	2188 (90.08%)	480 (87.43%)	​
Yes	310 (10.41%)	241 (9.92%)	69 (12.57%)	​
Hypertension	​	​	​	0.086
No	2493 (83.71%)	2020 (83.16%)	473 (86.16%)	​
Yes	485 (16.29%)	409 (16.84%)	76 (13.84%)	​
Renal insufficiency	​	​	​	0.113
No	2917 (97.95%)	2374 (97.74%)	543 (98.91%)	​
Yes	61 (2.05%)	55 (2.26%)	6 (1.09%)	​
Diabetic complications	​	​	​	**0.001**
No	2962 (99.46%)	2422 (99.71%)	540 (98.36%)	​
Yes	16 (0.54%)	7 (0.29%)	9 (1.64%)	​
Coronary artery disease	​	​	​	**0.004**
No	2877 (96.61%)	2335 (96.13%)	542 (98.72%)	​
Yes	101 (3.39%)	94 (3.87%)	7 (1.28%)	​
Chronic obstructive pulmonary disease	​	​	​	**0.037**
No	2958 (99.33%)	2409 (99.18%)	549 (100.00%)	​
Yes	20 (0.67%)	20 (0.82%)	0 (0.00%)	​
Asthma	​	​	​	0.066
No	2969 (99.70%)	2424 (99.79%)	545 (99.27%)	​
Yes	9 (0.30%)	5 (0.21%)	4 (0.73%)	​
Cardiac conditions, n (%)				
Cardiac insufficiency	​	​	​	**0.009**
No	2871 (96.41%)	2331 (95.97%)	540 (98.36%)	​
Yes	107 (3.59%)	98 (4.03%)	9 (1.64%)	​
Atrial fibrillation	​	​	​	0.107
No	2913 (97.82%)	2371 (97.61%)	542 (98.72%)	​
Yes	65 (2.18%)	58 (2.39%)	7 (1.28%)	​
Valvular insufficiency	​	​	​	0.221
No	2972 (99.80%)	2423 (99.75%)	546 (99.45%)	​
Yes	6 (0.20%)	6 (0.25%)	3 (0.55%)	​
Disease severity, n (%)				
Complex complicated condition	​	​	​	0.064
No	2797 (93.92%)	2272 (93.54%)	525 (95.63%)	​
Yes	181 (6.08%)	157 (6.46%)	24 (4.37%)	​
Critical condition	​	​	​	0.084
No	1034 (34.72%)	826 (34.01%)	208 (37.89%)	​
Yes	1944 (65.28%)	1603 (65.99%)	341 (62.11%)	​
Septic shock	​	​	​	0.121
No	2921 (98.09%)	2387 (98.27%)	534 (97.27%)	​
Yes	57 (1.91%)	42 (1.73%)	15 (2.73%)	​
Sepsis	​	​	​	0.113
No	2697 (90.56%)	2190 (90.16%)	507 (92.35%)	​
Yes	281 (9.44%)	239 (9.84%)	42 (7.65%)	​
Supportive care, n (%)				
Parenteral nutrition	​	​	​	**0.034**
No	2948 (99.99%)	2409 (99.18%)	539 (98.17%)	​
Yes	30 (1.01%)	20 (0.82%)	10 (1.82%)	​
Laboratory results				
WBC count (109/L), median (IQR)	10.27 [3.74; 44.53]	10.38 [3.71; 45.16]	9.54 [3.71; 42.35]	0.196
HGB (g/L), median (IQR)	90.00 [69.00; 113.00]	90.00 [69.00; 113.00]	91.00 [69.00; 114.00]	0.343
RBC count (1012/L), median (IQR)	3.00 [2.23; 3.79]	2.99 [2.22; 3.77]	3.01 [2.28; 3.85]	0.378
HCT (%), median (IQR)	28.10 [21.50; 35.30]	28.00 [21.50; 35.20]	28.50 [21.40; 35.80]	0.524
RDW-SD (fL), median (IQR)	52.30 [46.70; 59.70]	52.40 [46.80; 59.70]	51.70 [46.30; 59.00]	0.196
RDW-CV (%), median (IQR)	15.50 [14.10; 17.40]	15.50 [14.20; 17.40]	15.40 [14.00; 17.10]	0.162
MCV (fL), median (IQR)	95.00 [89.70; 100.70]	95.00 [89.70; 100.80]	94.70 [89.60; 100.30]	0.377
MCHC (g/L), median (IQR)	322.00 [311.00; 332.00]	321.00 [311.00; 332.00]	322.00 [312.00; 333.00]	0.093
MCH (pg), median (IQR)	30.60 [28.80; 32.80]	30.60 [28.70; 32.60]	30.80 [28.50; 32.80]	0.149
PLT count (109/L), median (IQR)	88.00 [30.00; 192.75]	88.00 [30.00; 188.00]	97.00 [28.00; 207.00]	0.373
PDW (fL), median (IQR)	12.30 [10.60; 14.70]	12.30 [10.60; 14.80]	12.30 [10.30; 14.00]	0.110
MPV (fL), median (IQR)	10.30 [9.50; 11.20]	10.30 [9.48; 11.20]	10.41 [9.51; 11.40]	0.232
Neutrophils percentage (%), median (IQR)	32.20 [17.50; 54.00]	32.20 [17.40; 53.50]	32.20 [17.60; 55.60]	0.434
Neutrophils count (109/L), median (IQR)	2.31 [1.12; 4.45]	2.31 [1.13; 4.55]	2.28 [1.06; 4.15]	0.136
Monocytes percentage (%), median (IQR)	8.00 [5.00; 12.80]	8.00 [5.00; 12.70]	8.05 [5.16; 13.40]	0.395
Monocytes count (109/L), median (IQR)	0.46 [0.30; 0.87]	0.46 [0.30; 0.88]	0.44 [0.28; 0.80]	0.190
Lymphocytes percentage (%), median (IQR)	36.70 [26.20; 48.70]	36.70 [26.60; 49.60]	36.70 [24.90; 46.70]	0.359
Lymphocytes count (109/L), median (IQR)	1.76 [1.25; 2.71]	1.76 [1.25; 2.74]	1.72 [1.20; 2.52]	0.199
Basophils percentage (%), median (IQR)	0.30 [0.20; 0.71]	0.30 [0.20; 0.71]	0.30 [0.21; 0.72]	0.428
Basophils count (109/L), median (IQR)	0.03 [0.01; 0.13]	0.03 [0.01; 0.13]	0.03 [0.01; 0.14]	0.340
Eosinophils percentage (%), median (IQR)	0.40 [0.10; 1.20]	0.40 [0.10; 1.20]	0.40 [0.10; 1.00]	0.833
Eosinophils count (109/L), median (IQR)	0.03 [0.01; 0.13]	0.03 [0.01; 0.14]	0.03 [0.01; 0.13]	0.509
Hs-CRP (mg/L), median (IQR)	10.75 [4.93; 20.28]	10.75 [5.09; 20.40]	10.70 [4.00; 19.30]	0.086
PCT (ng/mL), median (IQR)	0.11 [0.05; 0.20]	0.11 [0.04; 0.19]	0.11 [0.05; 0.22]	0.091
Urea (mmol/L), median (IQR)	5.12 [4.12; 6.25]	5.12 [4.15; 6.30]	5.10 [4.02; 6.04]	**0.022**
Creatinine (umol/L), median (IQR)	61.95 [50.00; 74.70]	61.95 [50.90; 74.80]	61.95 [49.50; 73.70]	0.123
UA (umol/L), median (IQR)	313.00 [247.00; 389.00]	313.00 [247.00; 390.00]	313.00 [244.00; 385.00]	0.381
CYSC (mg/L), median (IQR)	0.90 [0.80; 0.98]	0.90 [0.81; 0.99]	0.90 [0.77; 0.95]	**0.033**
TBIL (umol/L), median (IQR)	10.00 [7.40; 13.60]	10.00 [7.40; 13.80]	10.00 [7.40; 12.90]	0.205
DBIL (umol/L), median (IQR)	4.00 [3.00; 5.40]	4.00 [3.00; 5.40]	3.90 [2.90; 5.00]	0.062
AST (U/L), median (IQR)	19.60 [15.00; 27.00]	19.60 [15.00; 27.00]	19.20 [14.00; 26.00]	0.122
ALT (U/L), median (IQR)	15.00 [10.20; 24.30]	15.00 [10.10; 24.00]	15.00 [11.00; 26.10]	0.165
TBA (U/L), median (IQR)	5.00 [3.30; 8.00]	5.10 [3.40; 8.00]	4.80 [3.10; 7.50]	0.075
TP (g/L), median (IQR)	63.10 [59.70; 67.20]	63.10 [59.50; 67.20]	63.10 [60.20; 67.20]	0.305
ALB (g/L), median (IQR)	39.60 [36.60; 42.80]	39.60 [36.40; 42.70]	40.20 [37.10; 43.00]	**0.011**
AFU (U/L), median (IQR)	17.10 [14.00; 20.90]	17.10 [14.00; 21.00]	17.10 [14.10; 20.50]	0.445
CHE (U/L), median (IQR)	6097.00 [4881.00; 7556.75]	6097.00 [4831.00; 7489.00]	6221.00 [5134.00; 7856.00]	**0.003**
PA (mg/dL), median (IQR)	174.00 [135.62; 213.10]	174.00 [133.80; 212.00]	174.00 [141.00; 220.00]	**0.014**
α-HBDH (U/L), median (IQR)	242.50 [161.00; 419.00]	242.50 [161.00; 420.00]	240.50 [160.00; 401.00]	0.208
LDH (U/L), median (IQR)	298.00 [197.25; 537.00]	299.00 [198.00; 537.00]	296.00 [189.00; 520.00]	0.165
ALP (U/L), median (IQR)	78.00 [61.00; 102.00]	78.00 [62.00; 102.00]	79.00 [61.00; 105.00]	0.378
γ-GGT (U/L), median (IQR)	27.00 [17.00; 44.00]	27.00 [17.00; 44.00]	27.00 [18.00; 47.00]	0.337
ADA (U/L), median (IQR)	13.40 [9.30; 19.48]	13.40 [9.40; 19.60]	13.40 [9.00; 19.00]	0.421
TG (mmol/L), median (IQR)	1.30 [0.98; 1.78]	1.30 [0.97; 1.77]	1.30 [1.02; 1.85]	0.187
CHOL (mmol/L), median (IQR)	3.37 [2.76; 4.07]	3.37 [2.75; 4.06]	3.39 [2.78; 4.14]	0.489
TT (s), median (IQR)	16.40 [15.60; 17.50]	16.40 [15.60; 17.50]	16.40 [15.60; 17.40]	0.465
PT (s), median (IQR)	14.00 [12.80; 15.10]	14.00 [12.90; 15.10]	14.00 [12.80; 15.00]	0.203
Prothrombin time activity (%), median (IQR)	79.00 [69.05; 91.00]	79.00 [69.00; 90.00]	79.50 [70.00; 91.30]	0.194
INR, median (IQR)	1.12 [1.03; 1.22]	1.12 [1.03; 1.23]	1.12 [1.02; 1.21]	0.144
D-dimer (mg/L), median (IQR)	0.84 [0.41; 2.14]	0.84 [0.41; 2.13]	0.84 [0.38; 2.19]	0.405
Fibrinogen (g/L), median (IQR)	3.16 [2.53; 4.08]	3.16 [2.53; 4.13]	3.13 [2.50; 3.93]	0.161
APTT (s), median (IQR)	35.15 [29.60; 40.40]	35.15 [29.70; 40.40]	35.15 [29.50; 40.10]	0.135
Glucose (mmol/L), median (IQR)	5.00 [4.41; 5.95]	5.00 [4.42; 5.95]	5.00 [4.37; 5.94]	0.256
Phosphate (mmol/L), median (IQR)	1.25 [1.08; 1.43]	1.25 [1.08; 1.43]	1.26 [1.12; 1.44]	**0.034**
Sodium (mmol/L), median (IQR)	141.60 [139.70; 143.20]	141.60 [139.70; 143.20]	141.60 [139.90; 143.20]	0.552
Magnesium (mmol/L), median (IQR)	0.89 [0.84; 0.95]	0.89 [0.84; 0.95]	0.89 [0.83; 0.93]	0.057
Potassium (mmol/L), median (IQR)	3.92 [3.65; 4.20]	3.94 [3.67; 4.21]	3.90 [3.57; 4.10]	0.390
Calcium (mmol/L), median (IQR)	2.22 [2.14; 2.31]	2.22 [2.14; 2.31]	2.23 [2.16; 2.32]	**0.034**
Ferritin (ng/mL), median (IQR)	330.34 [273.25; 373.50]	330.34 [270.00; 378.00]	330.24 [303.47; 352.00]	0.232
CK (U/L), median (IQR)	40.00 [26.69; 60.00]	40.00 [27.00; 60.00]	40.00 [26.66; 58.00]	0.485

Bold values indicate statistical significance (P < 0.05).

Patients who developed severe pneumonia were younger and more likely to have ALL, complex complications, and critical illness. Notably, they exhibited the hallmark biological signatures of sepsis-induced cell death cascades: pronounced coagulation dysfunction (elevated INR, prolonged PT, elevated D-dimer), hepatocellular injury markers (elevated AST, ALP, ADA, α-HBDH, LDH) reflecting DAMP release from pyroptotic and apoptotic hepatocytes, and hematopoietic stress indices (reduced prealbumin, elevated phosphate) consistent with systemic metabolic reprogramming in sepsis (Table 2). These findings underscore that severe pneumonia in leukemia patients is accompanied by the systemic footprint of multiple programmed cell death modalities.

**TABLE 2:** Comparison of demographic characteristics and clinical characteristics between non-severe pneumonia and severe pneumonia patients in the training Cohort.

Characteristic	Total, N = 1700	Non-severe pneumonia, N = 1629	Severe pneumonia, N = 71	p-value
Age (year), median (IQR)	54.00 [36.00; 66.00]	55.00 [39.00; 66.00]	9.00 [4.00; 34.00]	**<0.001**
Gender, n (%)	​	​	​	0.254
Female	722 (42.47%)	697 (42.79%)	25 (35.21%)	​
Male	978 (57.53%)	932 (57.21%)	46 (64.79%)	​
Malignancy type, n (%)				
Acute myeloid leukemia	​	​	​	**<0.001**
No	787 (46.29%)	737 (45.24%)	50 (70.42%)	​
Yes	913 (53.71%)	892 (54.76%)	21 (29.58%)	​
Acute lymphoblastic leukemia	​	​	​	**<0.001**
No	1394 (82.00%)	1369 (84.04%)	25 (35.21%)	​
Yes	306 (18.00%)	260 (15.96%)	46 (64.79%)	​
Chronic lymphocytic leukemia	​	​	​	**0.032**
No	1541 (90.65%)	1471 (90.30%)	70 (98.59%)	​
Yes	159 (9.35%)	158 (9.70%)	1 (1.41%)	​
Chronic myeloid leukemia	​	​	​	**0.002**
No	1377 (81.00%)	1309 (80.36%)	68 (95.77%)	​
Yes	323 (19.00%)	320 (19.64%)	3 (4.23%)	​
Treatment history				
History of hematopoietic stem cell transplantation	​	​	​	0.149
No	1582 (93.06%)	1519 (93.25%)	63 (88.73%)	​
Yes	118 (6.94%)	110 (6.75%)	8 (11.27%)	​
History of corticosteroid use	​	​	​	1.000
No	1580 (92.94%)	1514 (92.94%)	66 (92.96%)	​
Yes	120 (7.06%)	115 (7.06%)	5 (7.04%)	​
History of G-CSF use	​	​	​	1.000
No	1607 (94.53%)	1539 (94.48%)	68 (95.77%)	​
Yes	93 (5.47%)	90 (5.52%)	3 (4.23%)	​
History of blood transfusion	​	​	​	1.000
No	1510 (88.82%)	1447 (88.83%)	63 (88.73%)	​
Yes	190 (11.18%)	182 (11.17%)	8 (11.27%)	​
History of myelosuppression	​	​	​	0.279
No	1556 (91.53%)	1494 (91.71%)	62 (87.32%)	​
Yes	144 (8.47%)	135 (8.29%)	9 (12.68%)	​
Comorbidities, n (%)				
Diabetes mellitus	​	​	​	0.226
No	1548 (91.06%)	1480 (90.85%)	68 (95.77%)	​
Yes	152 (8.94%)	149 (9.15%)	3 (4.23%)	​
Hypertension	​	​	​	**0.008**
No	1422 (83.65%)	1354 (83.12%)	68 (95.77%)	​
Yes	278 (16.35%)	275 (16.88%)	3 (4.23%)	​
Renal insufficiency	​	​	​	0.257
No	1657 (97.47%)	1586 (97.36%)	71 (100.00%)	​
Yes	43 (2.53%)	43 (2.64%)	0 (0.00%)	​
Diabetic complications	​	​	​	1.000
No	1695 (99.71%)	1624 (99.69%)	71 (100.00%)	​
Yes	5 (0.29%)	5 (0.31%)	0 (0.00%)	​
Coronary artery disease	​	​	​	0.522
No	1634 (96.12%)	1564 (96.01%)	70 (98.59%)	​
Yes	66 (3.88%)	65 (3.99%)	1 (1.41%)	​
Chronic obstructive pulmonary disease	​	​	​	1.000
No	1682 (98.94%)	1611 (98.90%)	71 (100.00%)	​
Yes	18 (1.06%)	18 (1.10%)	0 (0.00%)	​
Asthma	​	​	​	1.000
No	1696 (99.76%)	1625 (99.75%)	71 (100.00%)	​
Yes	4 (0.24%)	4 (0.25%)	0 (0.00%)	​
Cardiac conditions, n (%)				
Cardiac insufficiency	​	​	​	0.151
No	1618 (95.18%)	1553 (95.33%)	65 (91.55%)	​
Yes	82 (4.82%)	76 (4.67%)	6 (8.45%)	​
Atrial fibrillation	​	​	​	0.255
No	1658 (97.53%)	1590 (97.61%)	68 (95.77%)	​
Yes	42 (2.47%)	39 (2.39%)	3 (4.23%)	​
Valvular insufficiency	​	​	​	0.226
No	1694 (99.65%)	1624 (99.69%)	70 (98.59%)	​
Yes	6 (0.35%)	5 (0.31%)	1 (1.41%)	​
Disease severity, n (%)				
Complex complicated condition	​	​	​	**<0.001**
No	1578 (92.82%)	1534 (94.17%)	44 (61.97%)	​
Yes	122 (7.18%)	95 (5.83%)	27 (38.03%)	​
Critical condition	​	​	​	**<0.001**
No	566 (33.29%)	562 (34.50%)	4 (5.63%)	​
Yes	1134 (66.71%)	1067 (65.50%)	67 (94.37%)	​
Septic shock	​	​	​	0.390
No	1668 (98.12%)	1599 (98.16%)	69 (97.18%)	​
Yes	32 (1.88%)	30 (1.84%)	2 (2.82%)	​
Sepsis	​	​	​	0.217
No	1523 (89.59%)	1463 (89.81%)	60 (84.51%)	​
Yes	177 (10.41%)	166 (10.19%)	11 (15.49%)	​
Supportive care, n (%)				
Parenteral nutrition	​	​	​	1.000
No	1684 (99.06%)	1613 (99.02%)	71 (100.00%)	​
Yes	16 (0.94%)	16 (0.98%)	0 (0.00%)	​
Laboratory results				
WBC count (109/L), median (IQR)	10.55 [3.72; 46.23]	10.37 [3.71; 46.24]	16.68 [4.96; 42.22]	0.166
HGB (g/L), median (IQR)	90.00 [68.75; 112.00]	90.00 [69.00; 113.00]	80.00 [58.00; 99.50]	**0.001**
RBC count (1012/L), median (IQR)	2.99 [2.21; 3.74]	3.00 [2.22; 3.74]	2.74 [2.05; 3.46]	0.063
HCT (%), median (IQR)	28.00 [21.50; 35.02]	28.10 [21.60; 35.10]	25.50 [17.90; 31.60]	**0.001**
RDW-SD (fL), median (IQR)	52.45 [46.90; 59.70]	52.60 [47.00; 60.00]	48.90 [42.90; 54.75]	**<0.001**
RDW-CV (%), median (IQR)	15.50 [14.28; 17.40]	15.50 [14.30; 17.40]	15.10 [14.20; 16.35]	0.080
MCV (fL), median (IQR)	95.00 [89.70; 100.60]	95.20 [90.00; 100.90]	88.60 [84.20; 96.40]	**<0.001**
MCHC (g/L), median (IQR)	321.00 [311.00; 331.00]	321.00 [310.00; 331.00]	326.00 [316.00; 333.00]	**0.027**
MCH (pg), median (IQR)	30.50 [28.70; 32.50]	30.60 [28.80; 32.60]	28.80 [26.75; 30.65]	**<0.001**
PLT count (109/L), median (IQR)	88.00 [30.00; 184.00]	89.00 [31.00; 186.00]	58.00 [22.00; 118.50]	**0.008**
PDW (fL), median (IQR)	12.30 [10.60; 14.80]	12.30 [10.70; 14.80]	12.30 [10.05; 14.30]	0.196
MPV (fL), median (IQR)	10.30 [9.50; 11.20]	10.30 [9.50; 11.20]	10.20 [9.15; 10.80]	**0.047**
Neutrophils percentage (%), median (IQR)	32.20 [17.30; 53.20]	32.20 [17.60; 53.80]	21.80 [7.40; 32.20]	**<0.001**
Neutrophils count (109/L), median (IQR)	2.31 [1.15; 4.73]	2.31 [1.16; 4.73]	2.31 [1.02; 4.62]	0.632
Monocytes percentage (%), median (IQR)	8.00 [5.00; 11.93]	8.00 [4.90; 12.20]	8.00 [8.00; 10.55]	0.309
Monocytes count (109/L), median (IQR)	0.46 [0.30; 0.89]	0.46 [0.29; 0.90]	0.46 [0.46; 0.77]	0.224
Lymphocytes percentage (%), median (IQR)	36.70 [26.67; 49.85]	36.70 [26.50; 50.30]	36.70 [36.70; 43.95]	0.362
Lymphocytes count (109/L), median (IQR)	1.76 [1.26; 2.79]	1.76 [1.24; 2.80]	1.76 [1.76; 2.54]	0.105
Basophils percentage (%), median (IQR)	0.30 [0.20; 0.70]	0.30 [0.20; 0.70]	0.30 [0.20; 0.40]	0.177
Basophils count (109/L), median (IQR)	0.03 [0.01; 0.13]	0.03 [0.01; 0.13]	0.03 [0.01; 0.15]	0.942
Eosinophils percentage (%), median (IQR)	0.40 [0.10; 1.33]	0.40 [0.10; 1.40]	0.20 [0.10; 0.40]	**<0.001**
Eosinophils count (109/L), median (IQR)	0.03 [0.01; 0.14]	0.03 [0.01; 0.14]	0.02 [<0.01; 0.09]	0.128
Hs-CRP (mg/L), median (IQR)	10.75 [5.19; 20.42]	10.75 [4.51; 21.30]	10.75 [10.75; 10.75]	0.097
PCT (ng/mL), median (IQR)	0.11 [0.04; 0.19]	0.11 [0.04; 0.19]	0.10 [0.04; 0.12]	**0.027**
Urea (mmol/L), median (IQR)	5.12 [4.15; 6.32]	5.12 [4.20; 6.35]	4.18 [3.27; 5.30]	**<0.001**
Creatinine (umol/L), median (IQR)	61.95 [51.00; 74.93]	61.95 [51.70; 75.00]	41.00 [31.50; 61.98]	**<0.001**
UA (umol/L), median (IQR)	313.00 [248.75; 392.92]	313.00 [251.00; 391.00]	298.00 [223.50; 410.50]	0.180
CYSC (mg/L), median (IQR)	0.90 [0.81; 0.98]	0.90 [0.82; 0.98]	0.90 [0.67; 0.90]	**0.003**
TBIL (umol/L), median (IQR)	10.00 [7.30; 13.90]	10.00 [7.40; 13.90]	9.70 [5.95; 12.70]	**0.049**
DBIL (umol/L), median (IQR)	4.00 [3.00; 5.50]	4.00 [3.00; 5.50]	3.90 [2.60; 4.95]	0.173
AST (U/L), median (IQR)	19.60 [15.17; 28.00]	19.60 [15.00; 27.00]	29.30 [19.05; 49.90]	**<0.001**
ALT (U/L), median (IQR)	15.00 [10.20; 24.00]	15.00 [10.10; 23.80]	18.00 [12.45; 28.45]	0.057
TBA (U/L), median (IQR)	5.10 [3.30; 8.00]	5.10 [3.40; 7.90]	5.20 [2.95; 10.15]	0.527
TP (g/L), median (IQR)	63.10 [59.40; 67.40]	63.10 [59.50; 67.40]	62.90 [58.50; 66.40]	0.297
ALB (g/L), median (IQR)	39.60 [36.30; 42.80]	39.60 [36.30; 42.80]	39.20 [35.80; 42.35]	0.385
AFU (U/L), median (IQR)	17.10 [14.00; 21.00]	17.10 [14.00; 21.00]	18.70 [14.15; 23.15]	0.059
CHE (U/L), median (IQR)	6097.00 [4779.50; 7442.50]	6097.00 [4788.00; 7489.00]	5683.00 [4735.50; 6790.50]	0.109
PA (mg/dL), median (IQR)	174.00 [130.07; 210.55]	174.00 [133.00; 212.10]	139.00 [104.90; 174.00]	**<0.001**
α-HBDH (U/L), median (IQR)	242.50 [161.00; 435.25]	242.50 [158.00; 426.00]	341.00 [242.50; 772.00]	**<0.001**
LDH (U/L), median (IQR)	299.00 [199.00; 551.25]	299.00 [197.00; 537.00]	427.00 [299.00; 932.50]	**<0.001**
ALP (U/L), median (IQR)	78.00 [62.00; 103.00]	78.00 [62.00; 100.00]	114.00 [78.00; 168.00]	**<0.001**
γ-GGT (U/L), median (IQR)	27.00 [17.00; 43.00]	27.00 [17.00; 44.00]	22.00 [15.00; 38.00]	0.088
ADA (U/L), median (IQR)	13.40 [9.40; 20.00]	13.40 [9.20; 19.40]	18.00 [13.40; 43.55]	**<0.001**
TG (mmol/L), median (IQR)	1.30 [0.97; 1.78]	1.30 [0.96; 1.79]	1.30 [1.05; 1.54]	0.735
CHOL (mmol/L), median (IQR)	3.37 [2.76; 4.05]	3.37 [2.76; 4.07]	3.37 [2.79; 3.56]	0.262
TT (s), median (IQR)	16.40 [15.60; 17.60]	16.40 [15.50; 17.50]	16.70 [16.40; 17.95]	**0.003**
PT (s), median (IQR)	14.00 [12.90; 15.20]	14.00 [12.90; 15.20]	14.00 [13.90; 15.65]	**0.016**
Prothrombin time activity (%), median (IQR)	79.00 [69.00; 90.00]	79.00 [69.00; 90.40]	77.00 [62.80; 81.00]	**0.002**
INR, median (IQR)	1.12 [1.04; 1.23]	1.12 [1.03; 1.23]	1.15 [1.10; 1.28]	**0.004**
D-dimer (mg/L), median (IQR)	0.84 [0.41; 2.10]	0.84 [0.41; 2.08]	0.84 [0.72; 2.90]	**0.040**
Fibrinogen (g/L), median (IQR)	3.16 [2.50; 4.02]	3.16 [2.50; 4.02]	3.16 [2.54; 4.22]	0.804
APTT (s), median (IQR)	35.15 [29.80; 40.12]	35.15 [29.80; 40.20]	35.15 [29.50; 39.10]	0.485
Glucose (mmol/L), median (IQR)	5.00 [4.43; 5.95]	5.00 [4.42; 5.96]	5.00 [4.71; 5.70]	0.953
Phosphate (mmol/L), median (IQR)	1.25 [1.07; 1.43]	1.25 [1.07; 1.42]	1.42 [1.10; 1.56]	**0.006**
Sodium (mmol/L), median (IQR)	141.60 [139.70; 143.20]	141.60 [139.80; 143.20]	140.40 [138.55; 142.00]	**0.001**
Magnesium (mmol/L), median (IQR)	0.89 [0.84; 0.95]	0.89 [0.84; 0.95]	0.90 [0.83; 0.99]	0.324
Potassium (mmol/L), median (IQR)	3.94 [3.67; 4.21]	3.94 [3.67; 4.20]	4.05 [3.62; 4.31]	0.269
Calcium (mmol/L), median (IQR)	2.22 [2.13; 2.31]	2.22 [2.13; 2.31]	2.24 [2.13; 2.35]	0.163
Ferritin (ng/mL), median (IQR)	330.34 [278.80; 385.84]	330.34 [275.00; 387.00]	330.34 [330.34; 371.50]	0.128
CK (U/L), median (IQR)	40.00 [28.00; 61.00]	40.00 [28.00; 61.00]	40.00 [28.00; 72.00]	0.403

Bold values indicate statistical significance (P < 0.05).

### Independent predictors of sepsis risk

Univariate logistic regression revealed 32 variables demonstrating statistically significant relationships with severe pneumonia occurrence (P < 0.05). After multivariate logistic regression analysis, 9 factors independently associated with the risk of severe pneumonia in leukemic patients were identified: age, AML, CML, complex complicated condition, critical condition, RDW-CV, ALT, γ-GGT, and prothrombin time activity (Table 3). Notably, each of these predictors has a mechanistic link to sepsis-associated cell death pathways: coagulation parameters (prothrombin time activity) reflect immunothrombosis; hepatic enzymes (ALT, γ-GGT) signal hepatocyte injury from pyroptosis and necroptosis; and RDW-CV captures oxidative erythrocyte stress characteristic of ferroptosis-driven systemic inflammation.

**TABLE 3:** Univariate and multivariate analysis of risk factors for severe pneumonia in leukemia patients.

Characteristics	Univariate analysis						Multivariate analysis					
	Coef	SE	OR	95% CI	Z value	P value	Coef	SE	OR	95% CI	Z value	P value
Age	−0.060	0.005	0.942	0.932–0.952	−11.053	<0.001	−0.039	0.007	0.962	0.949–0.975	−5.471	**<0.001**
Acute myeloid leukemia	−1.098	0.228	0.334	0.210–0.515	−4.818	<0.001	−1.053	0.303	0.349	0.193–0.632	−3.473	**0.001**
Acute lymphoblastic leukemia	2.295	0.220	9.921	6.487–15.420	10.419	<0.001	​	​	​	​	​	​
Chronic lymphocytic leukemia	−1.614	0.718	0.199	0.033–0.634	−2.248	0.025	​	​	​	​	​	​
Chronic myeloid leukemia	−1.751	0.513	0.174	0.053–0.418	−3.411	0.001	−1.517	0.569	0.219	0.072–0.669	−2.668	**0.008**
Hypertension	−0.985	0.396	0.373	0.156–0.755	−2.487	0.013	​	​	​	​	​	​
Complex complicated condition	2.217	0.233	9.181	5.762–14.420	9.502	<0.001	0.704	0.297	2.021	1.130–3.614	2.373	**0.018**
Critical condition	1.794	0.372	6.011	3.090–13.520	4.826	<0.001	1.381	0.404	3.980	1.803–8.785	3.420	**0.001**
Sepsis	0.703	0.276	2.020	1.139–3.387	2.545	0.011	​	​	​	​	​	​
HGB	−0.013	0.004	0.987	0.980–0.994	−3.399	0.001	​	​	​	​	​	​
RBC count	−0.215	0.106	0.807	0.654–0.991	−2.030	0.042	​	​	​	​	​	​
HCT	−0.046	0.012	0.955	0.932–0.978	−3.711	<0.001	​	​	​	​	​	​
RDW-SD	−0.059	0.013	0.943	0.918–0.966	−4.524	<0.001	​	​	​	​	​	​
RDW-CV	−0.099	0.044	0.906	0.828–0.983	−2.246	0.025	−0.442	0.184	0.642	0.448–0.922	−2.402	**0.016**
MCV	−0.072	0.012	0.930	0.908–0.953	−5.966	<0.001	​	​	​	​	​	​
MCHC	0.015	0.006	1.015	1.003–1.027	2.440	0.015	​	​	​	​	​	​
MCH	−0.135	0.032	0.874	0.821–0.931	−4.223	<0.001	​	​	​	​	​	​
Neutrophils percentage	−0.023	0.005	0.977	0.967–0.987	−4.362	<0.001	​	​	​	​	​	​
Urea	−0.243	0.067	0.784	0.685–0.889	−3.622	<0.001	​	​	​	​	​	​
Creatinine	−0.043	0.006	0.958	0.946–0.969	−7.030	<0.001	​	​	​	​	​	​
CYSC	−0.810	0.388	0.445	0.194–0.882	−2.087	0.037	​	​	​	​	​	​
AST	0.003	0.001	1.003	1.000–1.005	2.534	0.011	​	​	​	​	​	​
ALT	0.003	0.001	1.003	1.000–1.006	2.049	0.040	−0.007	0.003	0.993	0.986–0.999	−2.139	**0.032**
PA	−0.009	0.002	0.991	0.988–0.995	−5.014	<0.001	​	​	​	​	​	​
AFU	0.030	0.012	1.031	1.004–1.055	2.455	0.014	​	​	​	​	​	​
ALP	0.004	0.001	1.004	1.003–1.006	5.799	<0.001	​	​	​	​	​	​
γ-GGT	0.003	0.001	1.003	1.002–1.005	4.027	<0.001	0.004	0.001	1.004	1.002–1.007	3.328	**0.001**
ADA	0.012	0.003	1.012	1.007–1.017	4.826	<0.001	​	​	​	​	​	​
Prothrombin time activity	−0.022	0.006	0.978	0.967–0.990	−3.626	<0.001	−0.057	0.017	0.944	0.914–0.976	−3.384	**0.001**
INR	0.737	0.348	2.090	0.984–3.944	2.117	0.034	​	​	​	​	​	​
Sodium	−0.133	0.029	0.876	0.828–0.928	−4.559	<0.001	​	​	​	​	​	​
Phosphate	0.752	0.316	2.121	1.127–3.890	2.381	0.017	​	​	​	​	​	​

Abbreviations: Coef, coefficient; SE, standard error; OR, odds ratio; CI, confidence interval; HGB, hemoglobin; RBC, red blood cell; HCT, hematocrit; RDW-SD, red blood cell distribution width-standard deviation; RDW-CV, red blood cell distribution width-coefficient of variation; MCV, mean corpuscular volume; MCHC, mean corpuscular hemoglobin concentration; MCH, mean corpuscular hemoglobin; CYSC, cystatin C; AST, aspartate aminotransferase; ALT, alanine aminotransferase; PA, prealbumin; AFU, alpha-L-fucosidase; ALP, alkaline phosphatase; γ-GGT, gamma-glutamyl transferase; ADA, adenosine deaminase; INR, international normalized ratio.

Bold values indicate statistical significance (P < 0.05).

### Nonlinear dose-response relationships

Restricted cubic spline analysis revealed significant nonlinear relationships between continuous predictors and sepsis risk (Table 4; Figure 2). Age demonstrated a threshold effect at 23.85 years (P < 0.001), with a protective association below this threshold (OR: 0.84 per year, 95%CI: 0.79–0.90) and no significant association above it—reflecting the disproportionate treatment intensity and resultant immune depletion in younger leukemia patients. γ-GGT exhibited a biphasic relationship with an inflection point at 14.00 U/L (P < 0.001), and ALT showed a threshold effect at 68.60 U/L (P = 0.028)—both thresholds likely demarcating the transition from compensated hepatocellular stress to decompensated DAMP-releasing cell death. RDW-CV and prothrombin time activity demonstrated stable linear inverse associations, consistent with their roles as continuous markers of inflammatory oxidative stress and coagulation cascade activation, respectively.

**TABLE 4:** Effect of Standardized Variables on severe pneumonia Risk: Odds Ratios from Segmented Logistic Regression Analysis.

Characteristics	OR per SD	95% CI	P value	P for overall	P for nonlinear
Age (<23.85 years)	0.84	0.79–0.90	<0.001	<0.001	<0.001
Age (≥23.85 years)	1.00	0.97–1.02	0.734		
RDW-CV (<14.60%)	1.19	0.76–1.85	0.449	0.025	0.371
RDW-CV (≥14.60%)	0.86	0.75–0.99	0.036		
ALT (<68.60 U/L)	1.01	1.00–1.03	0.105	0.040	0.028
ALT (≥68.60 U/L)	1.06	1.02–1.10	0.003		
γ-GGT (<14.00 U/L)	0.74	0.61–0.89	0.002	<0.001	<0.001
γ-GGT (≥14.00 U/L)	1.00	1.00–1.01	<0.001		
Prothrombin time activity (<79.00%)	1.00	0.98–1.03	0.764	<0.001	0.057
Prothrombin time activity (≥79.00%)	0.97	0.93–1.02	0.239		

Abbreviations: OR, odds ratio; SD, standard deviation; CI, confidence interval; RDW-CV, red blood cell distribution width-coefficient of variation; ALT, alanine aminotransferase; γ-GGT, gamma-glutamyl transferase.

**FIGURE 2:**
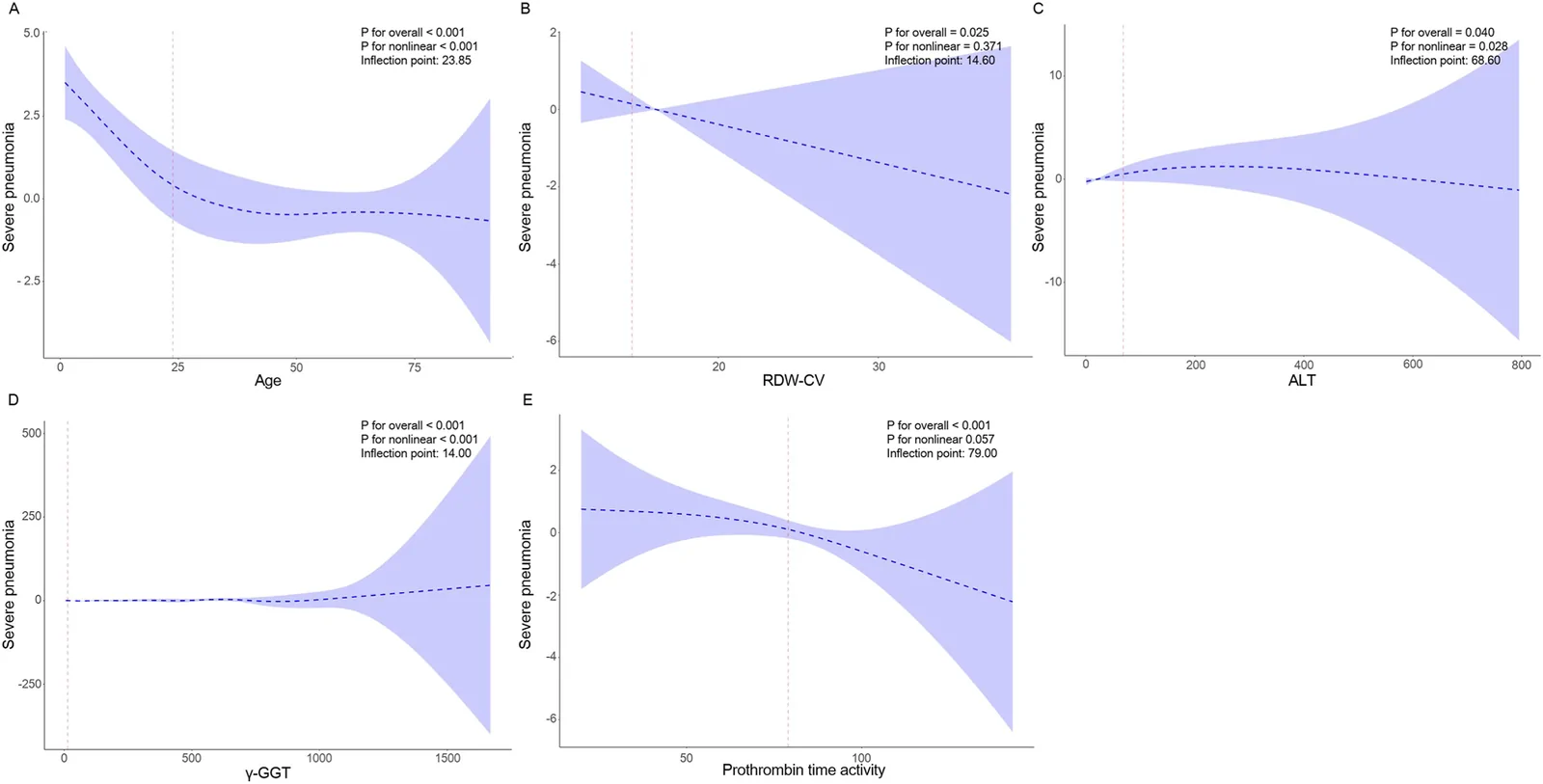
RCS analysis of the association between continuous predictor variables and the risk of severe pneumonia in leukemia patients. **(A)** Age showed a significant nonlinear relationship (P for nonlinear < 0.001), with an inflection point at 23.85 years. **(B)** RDW-CV demonstrated a linear association (P for nonlinear = 0.371). **(C)** ALT exhibited a nonlinear relationship (P for nonlinear = 0.028), with a threshold effect at 68.60 U/L. **(D)** γ-GGT showed a significant nonlinear relationship (P for nonlinear < 0.001), with an inflection point at 14.00 U/L. **(E)** Prothrombin time activity displayed a linear trend (P for nonlinear = 0.057). Abbreviations: RDW-CV, red blood cell distribution width-coefficient of variation; ALT, alanine aminotransferase; γ-GGT, gamma-glutamyl transferase.

### Feature selection and final predictors

LASSO regression identified 68 predictive variables (Supplementary Figures S1A,B). SVM-RFE identified ten key predictors: age, history of myelosuppression, ALL, cardiac insufficiency, atrial fibrillation, RDW-SD, prothrombin time activity, valvular insufficiency, TBA, and TP (Supplementary Figure S1C). Cross-validation analysis identified seven consistent predictive factors—age, history of myelosuppression, ALL, cardiac insufficiency, atrial fibrillation, RDW-SD, and prothrombin time activity (all VIFs <5; Supplementary Table S3)—which were incorporated into the final model. This constellation of predictors collectively maps the sepsis vulnerability landscape: immune depletion (myelosuppression), coagulation cascade engagement (prothrombin time activity), oxidative hematopoietic stress (RDW-SD), cardiopulmonary reserve (cardiac insufficiency, atrial fibrillation), and treatment-driven immune dysregulation (ALL, age).

### Model performance comparison

Eight machine learning methodologies were developed using ten rounds of 10-fold internal cross-validation. KNN demonstrated apparent overfitting (training AUC = 1.000), consistent with its absence of regularization mechanisms. During internal validation, logistic regression exhibited the strongest predictive performance (AUC = 0.828; Brier score = 0.106; sensitivity = 0.654; specificity = 0.853) (Table 5; Supplementary Figure S2). In external validation (n = 549), logistic regression preserved excellent predictive capacity (AUC = 0.801; Hosmer-Lemeshow P = 0.403), with decision curve analysis demonstrating clinical net benefit within probability thresholds of 0% to 81% (Figure 3; Supplementary Figure S3).

**TABLE 5:** Predictive performance comparison of the eight types of machine learning algorithms.

Model	Sensitivity	Specificity	PPV	NPV	Precision	Recall	F1 score	Balanced accuracy	Brier score
Train cohort									
Logistic regression	0.868	0.829	0.830	0.868	0.830	0.868	0.849	0.849	0.116
Decision tree	0.891	0.869	0.867	0.892	0.867	0.891	0.879	0.880	0.101
Random forest	0.991	0.975	0.974	0.991	0.974	0.991	0.983	0.983	0.018
XGBoost	0.974	0.966	0.965	0.975	0.965	0.974	0.969	0.970	0.028
SVM	0.878	0.823	0.826	0.875	0.826	0.878	0.851	0.850	0.116
KNN	1.000	1.000	1.000	1.000	1.000	1.000	1.000	1.000	0.013
LightGBM	0.947	0.918	0.917	0.947	0.917	0.947	0.932	0.932	0.057
Naive Bayes	0.869	0.810	0.814	0.865	0.814	0.869	0.841	0.839	0.131
Test cohort									
Logistic regression	0.654	0.853	0.142	0.985	0.142	0.654	0.233	0.754	0.106
Decision tree	0.654	0.902	0.198	0.986	0.198	0.654	0.304	0.778	0.095
Random forest	0.577	0.932	0.238	0.983	0.238	0.577	0.337	0.754	0.067
XGBoost	0.462	0.947	0.245	0.979	0.245	0.462	0.320	0.704	0.060
SVM	0.654	0.852	0.140	0.985	0.140	0.654	0.231	0.753	0.105
KNN	0.538	0.888	0.151	0.981	0.151	0.538	0.235	0.713	0.102
LightGBM	0.538	0.925	0.209	0.982	0.209	0.538	0.301	0.732	0.071
Naive Bayes	0.692	0.831	0.131	0.986	0.131	0.692	0.221	0.762	0.119

Abbreviations: PPV, positive predictive value; NPV, negative predictive value; SVM, support vector machine; KNN, k-nearest. neighbor; XGBoost, extreme gradient boosting; LightGBM, light gradient boosting machine.

**FIGURE 3:**
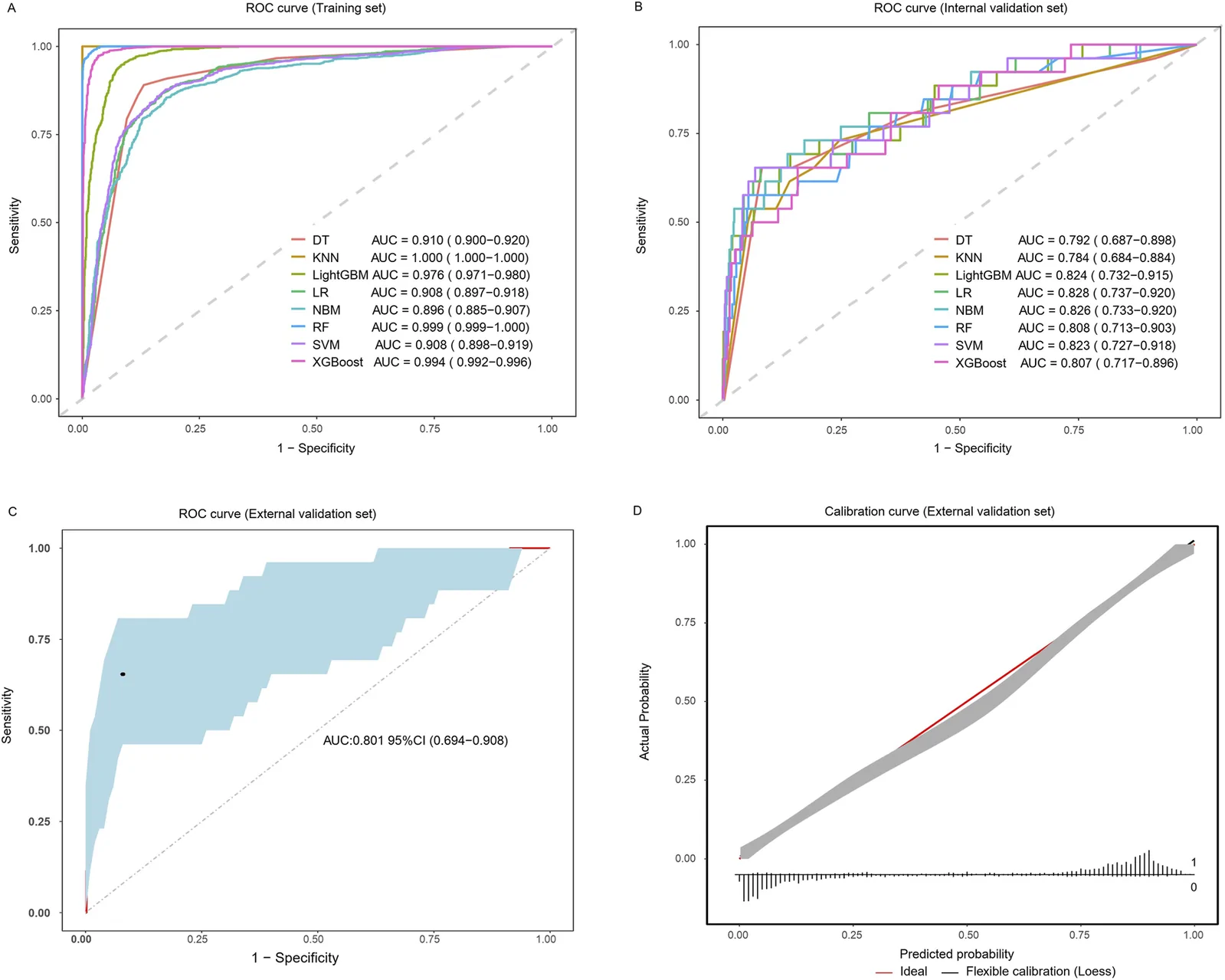
Performance comparison of machine learning algorithms for severe pneumonia prediction in hospitalized leukemia patients. **(A,B)** Receiver operating characteristic (ROC) curves comparing eight algorithms in the training **(A)** and internal validation **(B)** sets, with area under the curve (AUC) values and 95% confidence intervals listed for each model. **(C)** ROC curve of the final predictive model in the external validation set (AUC = 0.801, 95% CI: 0.694–0.908). **(D)** Calibration curve assessing the agreement between predicted probabilities and observed outcomes in the external validation set. Abbreviations: AUC, area under the curve; CI, confidence interval; DT, decision tree; KNN, k-nearest neighbor; LightGBM, light gradient boosting machine; LR, logistic regression; NBM, Naive Bayes model; RF, random forest; ROC, receiver operating characteristic; SVM, support vector machine; XGBoost, extreme gradient boosting.

### Final model and nomogram

Seven variables were statistically significant in multivariate logistic analysis (Table 6). The logistic equation was:

$$\text{logit}\left(P\right)=-4.614–0.065\times \text{Age}+1.106\times \text{Myelosuppression}+1.151\times \text{ALL}+2.348\times \text{Cardiac Insufficiency}+1.297\times \text{Atrial Fibrillation}-0.029\times \text{RDW}-\text{SD}-0.045\times \text{PTA}$$

**TABLE 6:** Multivariable logistic regression model for predicting severe pneumonia in the leukemic patients.

Characteristics	Multivariate analysis					
	Coefficients	SE	OR	95% CI	Z value	*P* value
Age	−0.065	0.003	0.937	0.937 (0.932–0.942)	−22.401	<0.001
History of myelosuppression	1.106	0.192	3.023	3.023 (2.073–4.409)	5.748	<0.001
Acute lymphoblastic leukemia	1.151	0.129	3.161	3.161 (2.455–4.070)	8.929	<0.001
Cardiac insufficiency	2.348	0.209	10.466	10.466 (6.948–15.764)	11.234	<0.001
Atrial fibrillation	1.286	0.304	3.618	3.618 (1.995–6.563)	4.233	<0.001
RDW-SD	−0.029	0.006	0.972	0.972 (0.961–0.983)	−4.956	<0.001
Prothrombin time activity	−0.045	0.004	0.956	0.956 (0.949–0.963)	−12.068	<0.001

Abbreviations: SE, standard error; OR, odds ratio; CI, confidence interval; RDW-SD, red blood cell distribution width-standard deviation.

These variables were incorporated into a nomogram for clinical risk stratification (Figure 4). Participants in the highest score quartile (total points: 186.94–262.13) had markedly elevated sepsis risk compared to lower quartiles (OR: 179.79, 95%CI: 121.46–266.12) (Supplementary Figures S4, S5).

**FIGURE 4:**
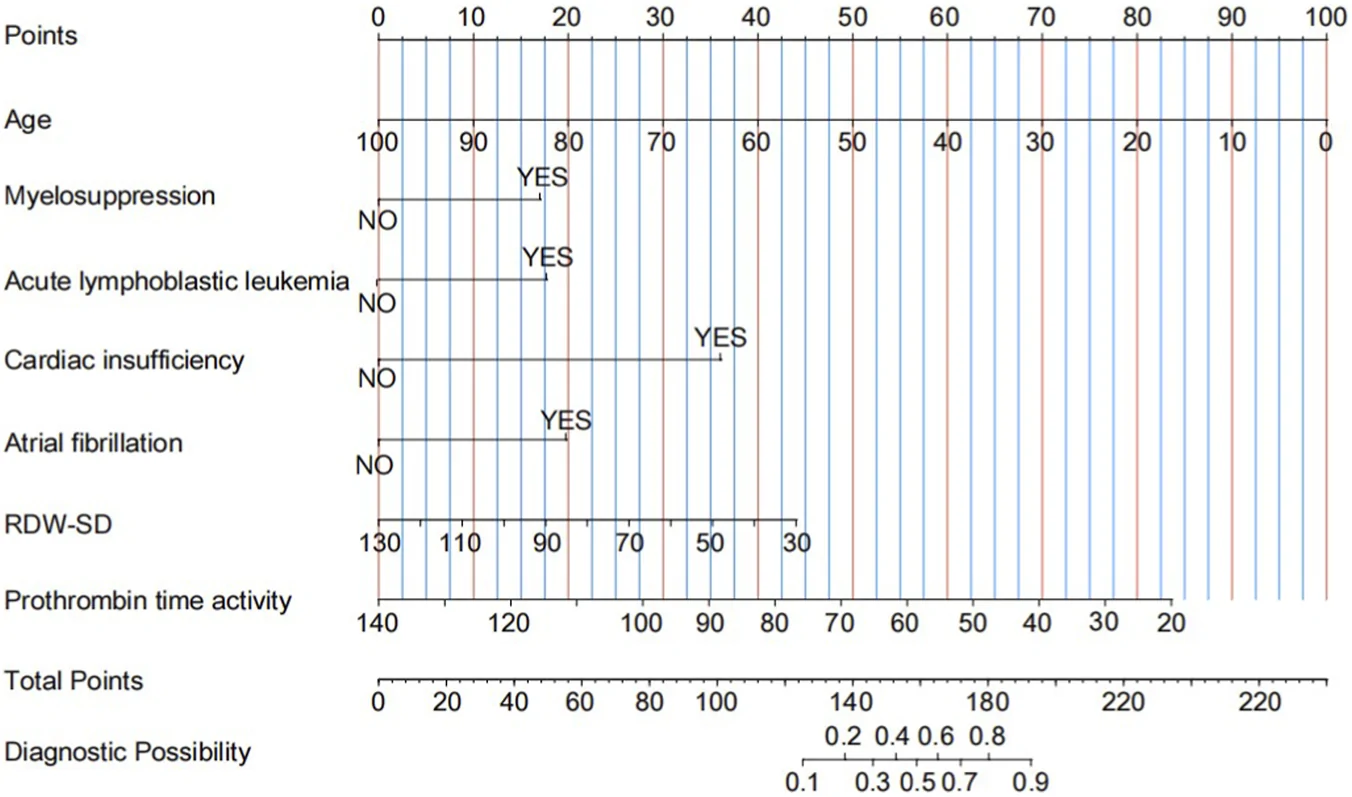
Nomogram for predicting the risk of severe pneumonia in hospitalized leukemia patients. The nomogram incorporates seven significant predictors identified by the machine learning model: age, history of myelosuppression, acute lymphoblastic leukemia status, cardiac insufficiency, atrial fibrillation, red blood cell distribution width-standard deviation (RDW-SD), and prothrombin time activity. Abbreviation: RDW-SD, red blood cell distribution width-standard deviation.

### SHAP-based feature attribution and mechanistic insight

SHAP analysis identified age, prothrombin time (PT) activity, and RDW-SD as the top predictors (mean |SHAP| ≈ 0.95, 0.65, and 0.30 respectively), with a significant interaction observed between age and RDW-SD (Figure 5). Young patients with high RDW-SD exhibited amplified sepsis risk, suggesting that age-related treatment intensity and concurrent oxidative erythrocyte stress synergistically drive immune vulnerability. Patient-level decomposition demonstrated that coagulation dysfunction and age together drove the majority of individual risk shifts, providing mechanistically transparent and clinically actionable explanations consistent with the immunothrombosis and immune depletion model of sepsis pathogenesis.

**FIGURE 5:**
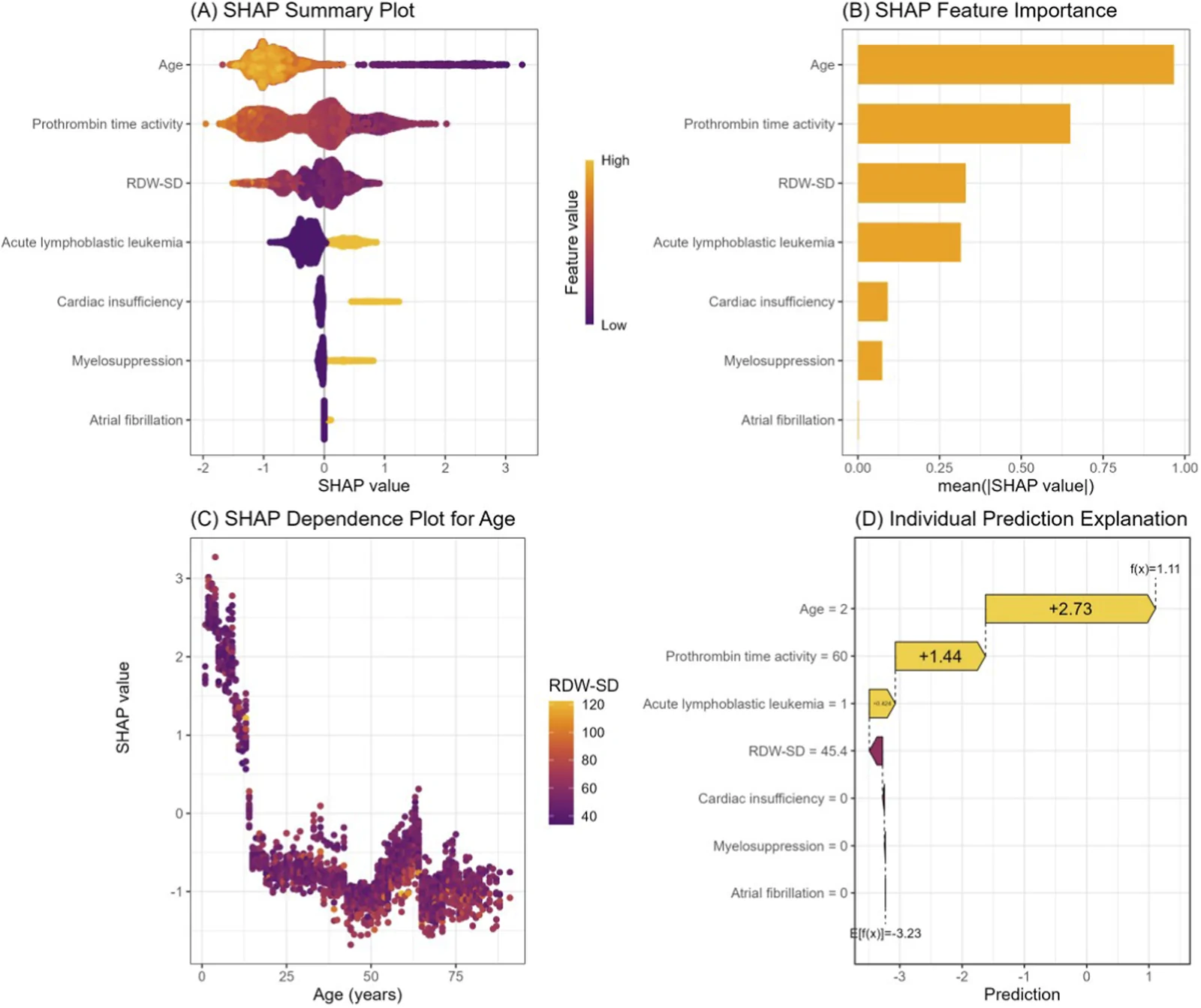
SHAP analysis of the predictive model. **(A)** SHAP summary plot showing the distribution of SHAP values across the top predictive features, colored by feature value (yellow = high, purple = low). **(B)** Bar plot of SHAP feature importance, quantified as the mean absolute SHAP value for each feature. **(C)** SHAP dependence plot for age, illustrating the relationship between age and its corresponding SHAP value, colored by RDW-SD to reveal interaction effects. **(D)** Individual prediction explanation waterfall plot, decomposing a single sample’s predicted outcome [f(x) = 1.11] into the contribution of each feature relative to the baseline expected value [E[f(x)] = –3.23].

### Sensitivity analysis

In the internal test cohort, the AUC of the model using MICE was 0.828 (95%CI: 0.763–0.890), comparable to complete case analysis AUC of 0.809 (95%CI: 0.749–0.872) (Supplementary Table S4; Supplementary Figure S6). In the external validation cohort, AUC values were 0.801 (95%CI: 0.751–0.854) for MICE and 0.794 (95%CI: 0.744–0.823) for complete case analysis (Supplementary Table S5; Supplementary Figure S7), confirming that model performance was not driven by imputation artifacts.

## Discussion

In this multicenter retrospective study, we developed and externally validated a prediction model for severe pneumonia in hospitalized patients with leukemia using routinely available clinical and laboratory data from 2,978 patients at two centers. The final LASSO–SVM-RFE pathway retained seven predictors—age, history of myelosuppression, ALL subtype, cardiac insufficiency, atrial fibrillation, RDW-SD, and prothrombin time activity—and logistic regression showed the most stable external discrimination (AUC = 0.801). These final predictors should be distinguished from variables identified in the exploratory multivariable logistic regression and RCS analyses, which addressed association rather than predictive feature selection.

Severe pneumonia in patients with leukemia may occur in a setting of profound disease- and treatment-related immune dysfunction (Arruga et al., 2020; Ehrlich et al., 2026; Ullah et al., 2025). In sepsis, dysregulated immunity, inflammasome signaling, immunosuppression, pyroptosis, and ferroptosis have been described in experimental and clinical literature (Wen et al., 2022; Liu et al., 2022; Boomer et al., 2011; Swanson et al., 2019; Venet and Monneret, 2018). These mechanisms provide a plausible biological context for severe infection, but the present study did not measure caspases, gasdermins, GPX4, lipid-peroxidation products, HMGB1, mitochondrial DNA, IL-1β, or IL-18. The model therefore predicts a clinical outcome from routine variables and does not demonstrate activation of any specific programmed cell-death pathway.

Prothrombin time activity was retained in the final prediction model. This finding is clinically plausible because infection-related inflammation and coagulation are closely linked, and sepsis-induced coagulopathy is well recognized (Levi and van der Poll, 2017; Iba et al., 2019). Coagulation abnormalities are also common in acute leukemia (Hisada et al., 2024). Nevertheless, prothrombin time activity in this dataset should be interpreted as a predictive clinical variable; the study did not directly measure tissue factor signaling, endothelial injury, microthrombi, or other components required to demonstrate immunothrombosis.

RDW-SD was also retained and ranked highly in the SHAP analysis. Previous studies have associated RDW with illness severity or adverse outcomes in sepsis and pneumonia (Moreno-Torres et al., 2022; Lee et al., 2013). However, RDW is a nonspecific hematologic index influenced by erythropoiesis, transfusion, nutritional status, inflammation, and other factors. Although ferroptosis is biologically relevant to sepsis in preclinical literature (Liu et al., 2022), RDW-SD should not be regarded as a direct ferroptosis biomarker in the absence of measurements such as iron-handling proteins, lipid-peroxidation products, or GPX4.

The exploratory multivariable and RCS analyses identified associations involving hepatic markers such as ALT and γ-GGT, but these variables were not retained by the final LASSO–SVM-RFE feature-selection pathway. Their RCS thresholds should therefore be interpreted as exploratory associations rather than determinants of individualized prediction. Liver dysfunction is biologically and clinically relevant in severe systemic infection (Strnad et al., 2017), but this study cannot attribute the observed hepatic laboratory changes to pyroptosis, necroptosis, DAMP release, or another specific cell-death mechanism.

History of myelosuppression remained a final predictor and is clinically consistent with the increased infectious vulnerability associated with treatment-related cytopenias and impaired host defense in hematologic malignancy (Ehrlich et al., 2026; Ullah et al., 2025). Likewise, leukemia subtype may capture differences in disease biology, treatment exposure, and immune status. These variables are useful for prediction, but the present data do not establish a specific causal immune-cell death mechanism.

Cardiac insufficiency and atrial fibrillation were retained as predictors, suggesting that reduced cardiovascular reserve may contribute to vulnerability during severe respiratory infection. Hemodynamic instability and impaired tissue perfusion are central features of critical infection and septic shock (Cecconi et al., 2018). However, the present model does not establish that these comorbidities increase risk through DAMP clearance, microthrombosis, or necroptotic injury; such mechanisms remain speculative.

The inverse association between age and risk requires particularly cautious interpretation. The observed pattern may reflect age-related differences in leukemia subtype, treatment selection or intensity, treatment phase, and treatment-related immune suppression rather than a direct biological protective effect of increasing age. Detailed chemotherapy regimen intensity, induction/consolidation/maintenance phase, and time since the most recent treatment were not available for complete adjustment. The reported age–RDW-SD SHAP interaction describes model behavior and should not be interpreted as proof of a causal age-dependent mechanism.

Methodologically, the study combined an explicit derivation/test split, training-only SMOTE, predictive feature selection, comparison of eight algorithms, internal testing, and independent external validation. The superior generalizability of logistic regression in this dataset also supports the practical value of an interpretable model for structured clinical data. At the same time, increasing the training event prevalence from 4.18% to 48.95% is aggressive oversampling and can affect probability calibration even when validation cohorts are left untouched. Accordingly, the external AUC and calibration statistics support discrimination and model fit at the observed validation prevalence, but absolute probabilities and any decision threshold require prospective recalibration and impact evaluation before routine clinical deployment. Transparent reporting and external validation remain central recommendations for prediction-model research (Collins et al., 2024; Collins and Moons, 2019).

This study has several limitations. First, the retrospective design introduces selection and measurement bias, although independent external validation provides an assessment of transportability. Second, the primary endpoint was ATS/IDSA-defined severe pneumonia, not Sepsis-3-defined sepsis; patients meeting only severe-pneumonia minor criteria cannot be assumed to have Sepsis-3 sepsis. Third, detailed chemotherapy regimen intensity, treatment phase, and time since treatment were not consistently available, which limits interpretation of the inverse age association. Fourth, pathogen-specific data and cytogenetic or molecular risk stratification were not incorporated. Fifth, direct biomarkers of programmed cell-death pathways were unavailable, so mechanistic interpretations are hypothesis-generating rather than demonstrated. Sixth, aggressive SMOTE oversampling may distort absolute-risk calibration, and prospective recalibration at real-world event prevalence is needed. Finally, variable-level missingness and center-to-center covariate differences should be reported in Supplementary Material to allow fuller assessment of imputation burden and transportability.

## Conclusion

In conclusion, we developed and externally validated an interpretable model for predicting severe pneumonia in hospitalized patients with leukemia. The seven final predictors—age, ALL subtype, history of myelosuppression, cardiac insufficiency, atrial fibrillation, RDW-SD, and prothrombin time activity—are routinely available and supported stable discrimination in internal and external validation. The model should be interpreted as a clinical prediction tool rather than evidence of specific programmed cell-death mechanisms. Because the endpoint was severe pneumonia and SMOTE substantially altered training prevalence, prospective validation, probability recalibration, and clinical-impact assessment are needed before routine use.

## Data Availability

The original contributions presented in the study are included in the article/Supplementary Material, further inquiries can be directed to the corresponding authors.
